# The Newer Ideas on Ventilation

**Published:** 1915-09

**Authors:** 


### THE NEWER IDEAS ON VENTILATION.

We do not ventilate in order to increase the oxygen and diminish the carbon dioxide in the air, says *Life and Health.* The old notion of ventilating in order to alter the chemical composition of the air has been pretty thoroughly exploded. Dr. Luther H. Gulick gave a terse statement of the new view at the School of Hygiene Congress at Buffalo:

"It used to be assumed that the exact percentage of oxygen in the air was an important factor in determining the quantity of this element absorbed and used by the body. We now know that within certain rather wide limits the percentage of oxygen has nothing to do with the case. The 'factor of safety' (Meltzer) in the functioning of the oxygen-taking and oxygen-carrying apparatus is such that under any of the conditions found in ordinary life the amount of oxygen taken in and consumed is determined solely by the demands of the body, and not by the percentage in the air; that is, a horse can not drink any more out of a lake than he can out of a trough. The experimental data referred to show that the oxygen consumption of the body is not in any way affected by lessening the oxygen in the air till it has been reduced from 21 per cent., where it is normally, to about 15 per cent.

"We also know that such lowering of oxygen is never found except under the controlled conditions of the laboratory. In other words, a tightly shut schoolroom full of pupils without any artificial ventilation will not suffer from lack of oxygen. They probably will suffer, but not from oxygen starvation. The exchange of gases through cracks in doors and windows, as well as through walls, floors and ceilings, is so rapid as to maintain a practically uniform atmospheric balance in gases."

The present notion is that air is contaminated by the gases given off from the body, through the skin, lungs and natural outlets for excretions.—Editor, *Register*.

